# Challenges in implementing treat-to-target in rheumatoid arthritis: a perspective from Brazilian rheumatologists

**DOI:** 10.1186/s42358-024-00403-w

**Published:** 2024-08-26

**Authors:** Adriana Maria Kakehasi, Angela Luzia Branco Pinto Duarte, Claiton Viegas Brenol, Diogo Souza Domiciano, Ieda Maria Magalhães Laurindo, Karina Rossi Bonfiglioli, Licia Maria Henrique da Mota, Maya H. Buch, Eduardo de Almeida Macêdo, Ricardo Machado Xavier

**Affiliations:** 1 Universidade Federal de Minas Gerais, Avenida Alfredo Balena, 190, Belo Horizonte, Brazil; 2 Universidade Federal de Pernambuco, Recife, Brazil; 3 Universidade Federal do Rio Grande do Sul, Porto Alegre, Brazil; 4 Universidade de São Paulo, São Paulo, Brazil; 5 Universidade Nove de Julho, São Paulo, Brazil; 6 Universidade de Brasília, Brasília, Brazil; 7 NIHR Leeds Musculoskeletal Biomedical Research Unit, University of Leeds, Leeds, UK; 8 AbbVie Farmacêutica Ltda, São Paulo, Brazil

**Keywords:** Rheumatoid arthritis, Treat-to-target, Patient-reported outcomes, Online surveys

## Abstract

**Background:**

Patient management in rheumatoid arthritis (RA) has evolved to a “treat-to-target” (T2T) approach, which entails intensive treatment and regular follow-up with the goal of achieving low levels of disease activity or clinical remission. Even though a T2T approach is endorsed by professional organizations and yields superior outcomes, its implementation remains incomplete. EVEREST (EleVatE care in RhEumatoid arthritiS with Treat-to-target) is a quality-improvement initiative designed to improve the widespread implementation of a personalized T2T strategy and enable patients with RA to reach their full potential for remission. We describe the Brazilian results from the Global T2T Survey, first part of the EVEREST program.

**Methods:**

Between June and September 2022, we conducted an online survey targeting rheumatologists in Brazil. Our objective was to evaluate the barriers and knowledge gaps hindering the effective implementation of T2T strategies. To achieve this, we employed a set of multiple-choice questions specifically crafted to elicit responses categorized in a structured order.

**Results:**

166 rheumatologists participated in the survey, 51% of them with more than 21 years of experience in rheumatology. Regarding the perceived challenges in the management of RA in clinical practice, the highest percentage of agreement/strong agreement among the participants was related to the contradictory results of disease activity measures (60%). In terms of the main barriers to assess the disease activity in clinical practice, the lack of adherence to treatment and contradictory assessments between patient-reported outcomes and composite measures were indicated by 75% and 59% of the participants, respectively, as a moderate/serious barrier. The most frequently knowledge and skill gaps related to the management of RA pointed out by the participants were on the difficulty to assess patients’ health literacy (54% stated to have no more than intermediate knowledge on standardized methods to assess it and 43% no more than intermediate skills on determining the level of health literacy of the patients). In general, the use of tools to support the management of RA patients in clinical practice was indicated to be unusual by the participants. Self-reflection questionnaires, patient education materials and treatment consideration checklists were pointed out as the least frequently used tools (85%, 64% and 62% of the participants stated to use them never, rarely, or only sometimes, respectively).

**Conclusions:**

Our findings indicate a greater need for design, selection, and uptake of practical strategies to further improve communication between healthcare providers and patients with RA, as well as for promoting well-informed, collaborative decision-making in their care.

**Supplementary Information:**

The online version contains supplementary material available at 10.1186/s42358-024-00403-w.

## Introduction

Rheumatoid arthritis (RA) is one of the most prevalent rheumatic immune-mediated diseases, affects 0.5–1% of the worldwide population, and causes considerable impact on the quality of life of individuals [1, 2]. Moreover, RA is associated with high morbidity and increased mortality, thus posing a significant economic burden to society [2]. Over the past two decades, the treatment of RA patients has evolved considerably, due to the introduction of new therapies and advances in the management of the disease [1]. As an important part of these advances, the use of a “treat-to-target” (T2T) approach has been established as a core strategy to achieve better outcomes in RA treatment and encompasses elements such as intensive treatment and regular follow-up, with the goal of enabling more patients to achieve low levels of disease activity or clinical remission [3, 4]. The concept of clinical remission as the primary treatment goal in RA has gained greater significance. While it was previously seen as an ambitious goal, current treatments now allow a substantial proportion of patients to attain remission [4–7].

Even though a T2T approach yields superior outcomes and has been endorsed by professional organizations, its implementation remains challenging and incomplete in clinical practice [8–10]. Barriers to adequate implementation can include knowledge gaps regarding treatment efficacy or safety, physician or patient preference and adherence patterns, and regional constraints to resources, access, and reimbursement [9, 11, 12]. Therefore, it is important to assess such barriers both globally and regionally.

EVEREST (EleVatE care in RhEumatoid arthritiS with Treat-to-target) is an initiative developed by AbbVie and led by a global executive steering committee composed of a group of experts in RA. The overarching goals of EVEREST are (1) to enable health-care professionals to understand and overcome their barriers to T2T implementation, (2) to allow patients to maximize their potential in achieving or maintaining clinical remission, and (3) to enable patients and their providers to speak a common language and increase their willingness to adjust treatments when needed. The initiative is based on implementation science and comprises three key phases planned to be implemented over a 6-year period. The first phase, which took place between 2021 and 2022, aimed at understanding barriers to and facilitators of T2T implementation through a comprehensive literature review and narrative synthesis, as well as through analysis of initiatives which have shown in published studies a potential to optimize that implementation in clinical practice. The results of the literature searches have been presented in abstract form and have informed subsequent steps [13]. The next initiative, aiming to capture the perception of rheumatologists, was the Global T2T survey, which was based on a conceptual framework used for strategic planning of continuing education activities designed for clinicians and their interprofessional healthcare teams [14–16]. The Global T2T Survey enlisted participation of rheumatologists from around the world. The present article outlines the findings derived from the Global T2T Survey pertaining to Brazilian rheumatologists.

## Methods

### Implementation of EVEREST in Brazil—the global T2T survey

Under guidance from the global executive steering committee, a local executive steering committee was established, comprising active and academic rheumatologists based in Brazil. Collaborative advisory board meetings were convened with these experts to delve into the understanding of local requirements and nuances, with the goal of tailoring EVEREST’s global initiatives to suit the Brazilian context (the survey is presented in the Supplementary Materials). As part of this endeavor to grasp the local landscape, Brazilian rheumatologists were invited to participate in the Global T2T survey, which was conducted online between June and September 2022. The survey was distributed to rheumatologists across 36 countries and garnered responses from those with a minimum of 2 years of active practice following fellowship, encompassing over 10 distinct RA patients each year, and possessing knowledge of T2T criteria. With regard to participants from Brazil, the survey was advertised during local congresses and medical events, as well as through an invitation sent to a list of approximately 1600 rheumatologists. Participation was both anonymous and voluntary, and participants were not provided compensation. Barriers to implementation of T2T were assessed in several ways through nine questions on different aspects related to clinical practice. Firstly, participants were asked to rate their level of agreement with statements related to the care of patients with RA, both regarding general issues in clinical practice and specific challenges to T2T implementation. Participants were then tasked with assessing the degree to which specific factors served as barriers to T2T implementation, including self-reported knowledge and skill gaps, frequency of patient monitoring, and use of tools related to decision-making. The current analysis outlines the outcomes of the survey within the Brazilian participant cohort.

### Statistical analysis

Descriptive analyses were undertaken, without formal comparisons between different groups. The survey included multiple-choice questions designed to elicit ordered categorical responses using Likert-like scales. These responses provided a more nuanced comprehension and interpretation of the knowledge gaps, as well as the perceived barriers and facilitators concerning the implementation of the T2T approach.

## Results

### Participant profile

A total of 166 rheumatologists from Brazil (from a total of 903 participants globally) participated in the online survey. The professional profile of these individuals is summarized in Table 1. 51% of the participants had more than 21 years of experience in rheumatology, and 68% had 6 or more years of experience with applying the T2T approach in their practice. The majority (72%) of respondents reportedly apply a T2T strategy to at least 50% of their patients with RA.

**Table 1:** Professional profiles of respondents of the online survey (*N* = 166)

Characteristic	Frequency
Years of experience practicing rheumatology	
2 to 10	19%
11 to 21	30%
More than 21	51%
Years of experience applying the T2T approach	
Up to 2	7%
3 to 5	25%
6 to 10	32%
10 or more	36%
Percentage of patients to whom a T2T approach is applied	
Zero	4%
Less 25%	11%
25 to 50%	13%
50 to 75%	25%
More than 75%	47%

*T2T* treat-to-target

### Challenges and barriers in clinical practice

Table 2 displays the responses to perceived challenges in clinical practice, which indicated general agreement with the statements. For example, there was agreement or strong agreement ranging from 42 to 60% with statements about the challenges to assess disease activity. Some of these barriers are direct (e.g., “I find it challenging to identify patient preferences to help determine an appropriate treatment target”), whereas others may be considered indirect (e.g., “Disease activity measures can give contradictory results”) or circumstantial (such as “I find it challenging to use T2T approach with patients on telehealth appointments”). Table 3 shows the responses to perceived barriers to assessing disease activity in clinical practice. The barriers most frequently cited pertain to patient adherence and to conflicting evaluations between patient-reported outcome (PRO) measures and composite measures. Moreover, the results show that 53% of respondents considered as a moderate or severe barrier to assessing disease activity the availability of insufficient financial resources to allow seeing patients frequently enough to meet T2T recommendations, while 49% considered that medical charts are not adapted to document measures.

**Table 2:** Perceived challenges in clinical practice (*N* = 166)

Statement	Slight agreement	Agreement/strong agreement
Disease activity measures can give contradictory results	21%	60%
In my practice, I only use composite measures to assess disease activity	15%	59%
I find it challenging to use T2T approach with patients on telehealth appointments	19%	54%
Patients’ perspectives on what constitutes successful treatment outcomes often differs from my own perspectives	27%	43%
I find it challenging to identify patient preferences to help determine an appropriate treatment target	19%	49%
I find it challenging to monitor disease activity	16%	42%

*T2T* treat-to-target

**Table 3:** Perceived barriers to assessing disease activity in clinical practice (*N* = 166)

Statement	Moderate barrier	Serious barrier
Change in disease activity potentially due to patient not adhering to treatment	31%	44%
Contradictory assessments between patient-reported outcomes and composite measures	42%	17%
Patient skipping too many regular appointments	23%	31%
Insufficient financial resources do not allow me to see patients frequently enough to meet T2T recommendations	29%	24%
Medical records or charts are not adapted to document these measures	36%	16%

*T2T* treat-to-target

### Gaps in knowledge, skills, and action

**Fig. 1:**
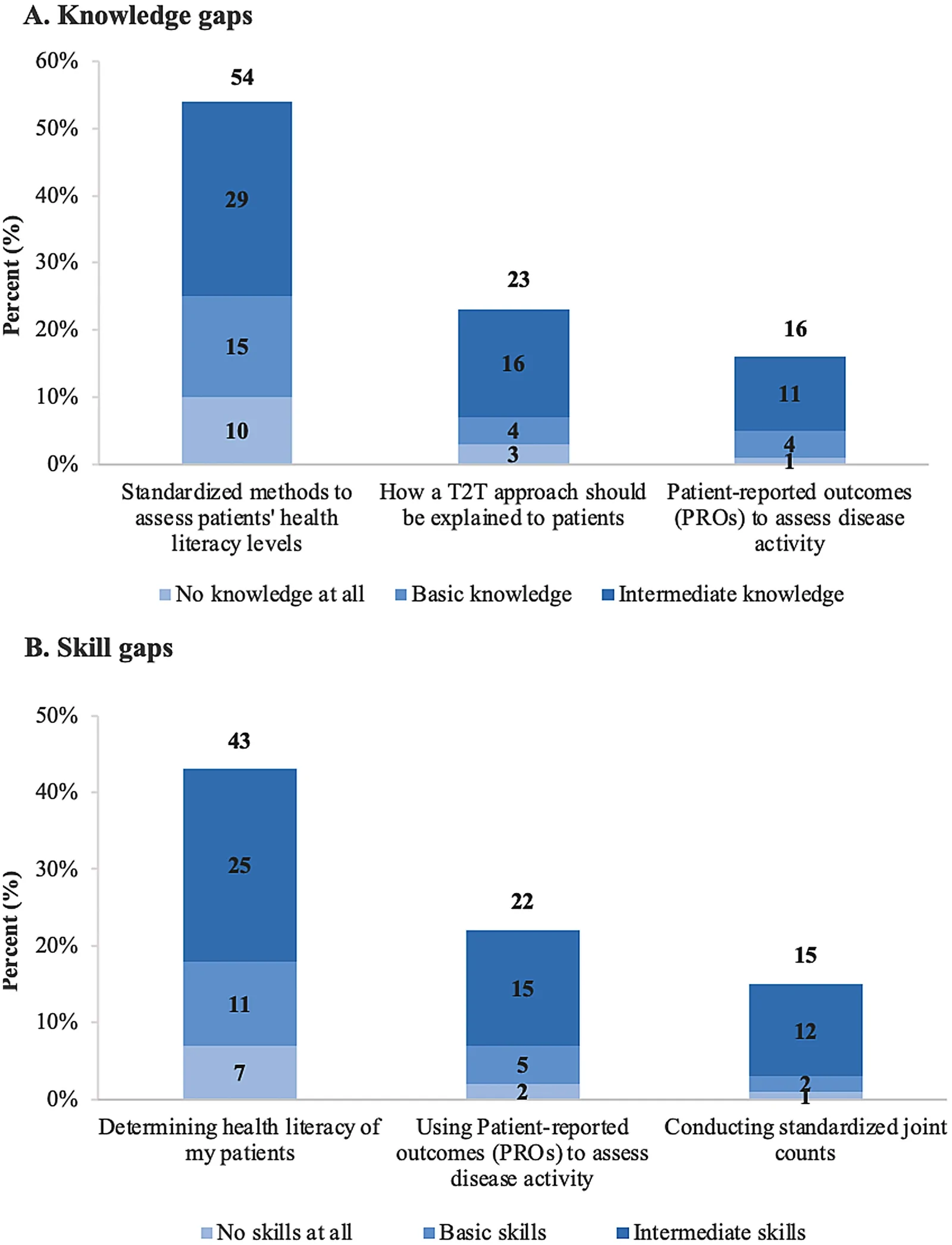
Self-reported knowledge (Panel A) and skill (Panel B) gaps linked to T2T implementation (*N* = 166). Shown are percentages for each category (inside bars) and overall (on top of bars)

Figure 1 showcases the leading three areas of knowledge (Panel A) and skill (Panel B) gaps that participants identified in their self-assessed proficiency regarding tasks linked to implementation of the T2T approach. Notably experienced with T2T practices, the rheumatologists surveyed here expressed reservations about their knowledge and skills concerning evaluating health literacy, employing PRO measures for disease activity assessment, and conducting standardized joint counts. For example, at best intermediate knowledge was reported by 22% of respondents regarding the use of PRO measures and 15% regarding standardized joint counts.

**Fig. 2:**
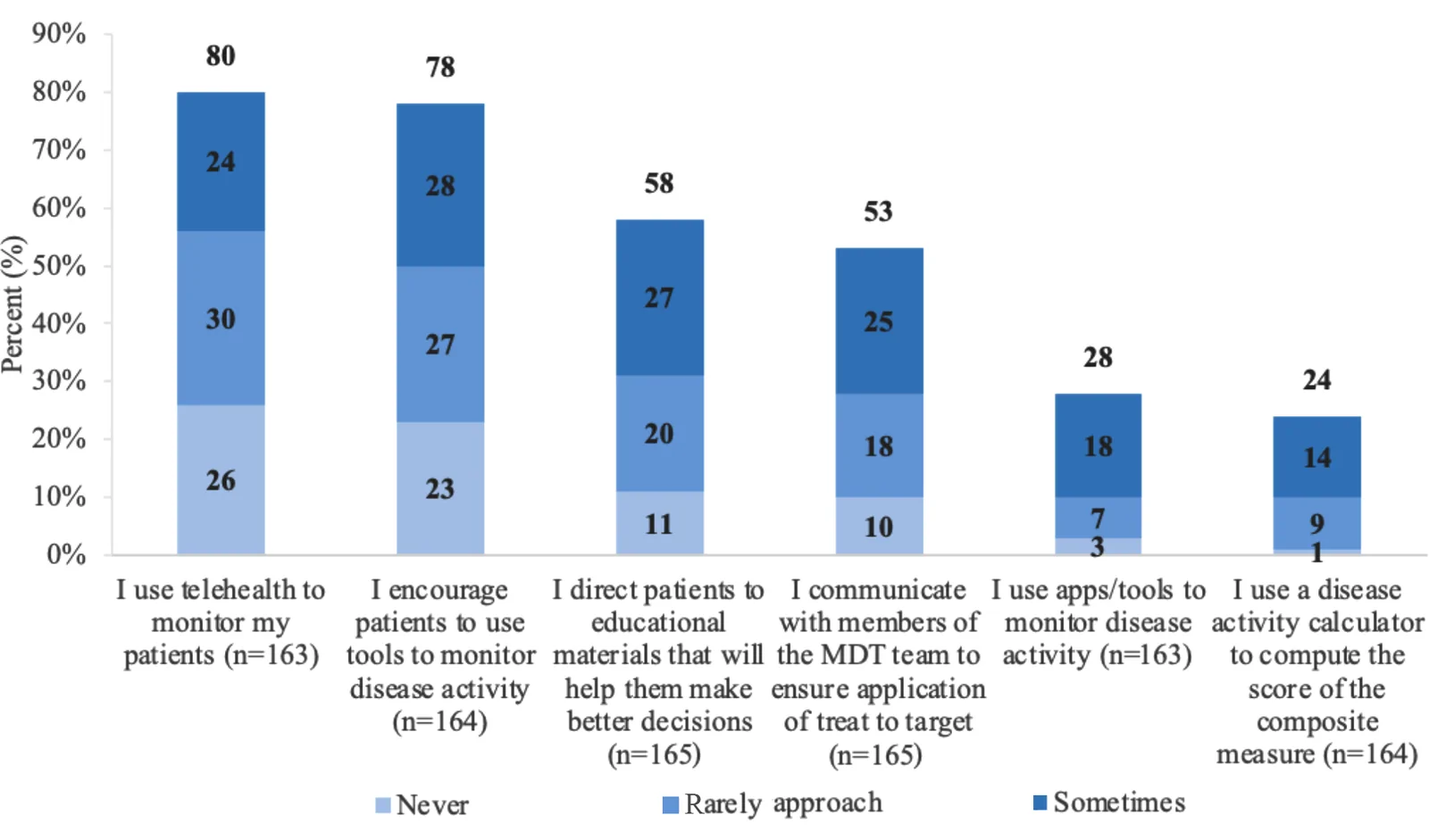
Reported frequency of tasks related to monitoring of disease activity (*N* = 166). Shown are percentages for each category (inside bars) and overall (on top of bars)

**Fig. 3:**
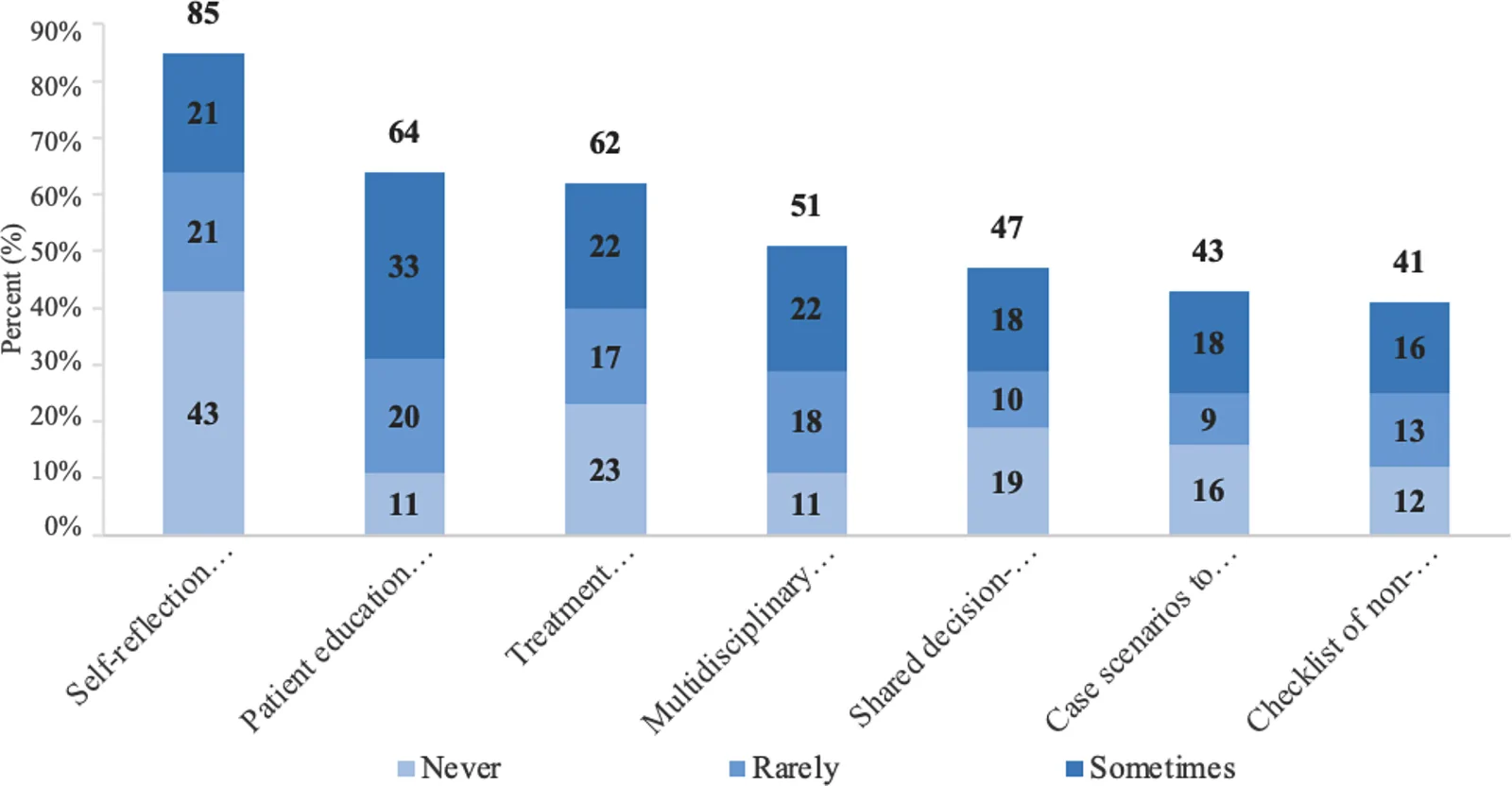
Reported frequency of use of tools related to decision-making (*N* = 166). Shown are percentages for each category (inside bars) and overall (on top of bars)

Figure 2 presents the reported frequency of use of tasks associated with monitoring of disease activity. The data suggests that tools necessitating active patient involvement are the least utilized, as opposed to those primarily employed by rheumatologists. Lastly, Fig. 3 portrays the reported frequency of use of tools related to decision-making. Among the least frequently used tools were self-reflection questionnaires, patient education materials, and treatment consideration checklists. When inquired about the reasons behind the non-utilization of such tools, prevailing factors included unawareness of their existence, perceived time requirements for their use, and concerns regarding cultural adaptation (specific data not displayed).

## Discussion

Our results show that there is general agreement regarding commonly perceived barriers to T2T implementation in clinical practice, and these include reliable elicitation of patient preferences, use and interpretation of disease activity measures, and challenges related to telehealth. Moreover, gaps in knowledge, skills, and action as related to assessment of health literacy, the correct use of PRO measures, and the conduct of standardized joint counts seem to underlie some of the difficulties in implementing T2T. Of note, the least used tools to assess disease activity are those involving active patient participation, as opposed to those primarily employed by rheumatologists. Finally, self-reflection questionnaires, patient education materials, and treatment consideration checklists seem to be seldom utilized by Brazilian rheumatologists. Of note, the current results are qualitatively similar to those from the worldwide experience within the EVEREST initiative [17]. Possible exceptions to this are nominally lower frequencies of use among Brazilian rheumatologists than worldwide of apps/tools to monitor disease activity and of disease activity calculators (among tasks described in Fig. 2), as well as of case scenarios to improve decision-making (among tools described in Fig. 3). On the other hand, among the perceived barriers described in Table 3, although the overall distribution was similar between Brazilian and worldwide rheumatologists, the former rated many of them as serious more frequently than the latter.

Despite the available evidence that a T2T strategy can improve symptoms and decrease long-term disease progression, many patients living with RA do not achieve guideline-recommended T2T goals [8–10, 18, 19], something that can lead to increased morbidity and mortality [12, 20]. In the US, for example, routine use of quantitative measurement for patients with RA remains suboptimal despite evidence of increase over time [19]. In a study from Brazil involving 1115 patients with RA from 11 centers, with a median disease duration of over 10 years, it was found that nearly half of the patients failed to achieve T2T goals and 55.2% developed erosive disease. Other notable findings from Brazil were the frequent use of corticosteroids and a delay in initiating disease-modifying anti-rheumatic drugs (DMARDs) [10]. It should be noted that this was a cohort with established disease, therefore indicating the consequences of diagnostic and therapeutic constraints which may be improved by a T2T strategy. Qualitatively similar findings to those from Brazil have been reported in several other countries [8, 18, 21–23].

Effective implementation of a T2T strategy in routine clinical practice relies on a number of factors including an understanding and willingness to adopt the principles of T2T by healthcare professionals and patients [12, 24]. Coupled with the availability of resources, this commitment may depend on proper awareness and education of those players. There is evidence that educational strategies can improve physician knowledge of and agreement with the T2T recommendations in RA [25]. Likewise, patient education is paramount to support change in RA treatment when recommended by a rheumatologist [26]. Nevertheless, one of the conclusions from the first phase of EVEREST was that although interventions designed to improve T2T implementation are available, there is as yet limited evidence for their direct impact toward that goal [13]. Moreover, education strategies and tools should be designed based on perceived gaps reported by healthcare professionals and patients. Likewise, the effectiveness and feasibility of these strategies likely vary according to regional features and healthcare settings [13]. Therefore, initiatives such as the one embodied in EVEREST have the potential to considerably improve T2T implementation in a manner that takes into account regional needs. That said, even reported adherence to guideline recommendations does not always equate to actual implementation of such recommendations, a reason why assessment of the effectiveness constitutes the planned third phase of EVEREST [27].

In the present study, it becomes evident that even experienced rheumatologists who possess self-reported knowledge and practical experience with applying the T2T strategy face various obstacles when attempting to effectively integrate this strategy into their clinical practice. One of these barriers relates to managing contradictory assessments between PRO measures and composite measures**—**a well-recognized concern within the rheumatology community [28]. This concern may be attributed, in part, to the frequent disparity between patient assessments and those conducted by physicians, a phenomenon documented in several studies. Notably, patients often assign higher scores than physicians in these assessments [28–30]. A related issue pertains to potential contradictions between the subjective and the objective components of composite measures assessed by physicians [31]. Numerous instances arise where different aspects of disease activity evaluation hold distinct significance for patients and for medical professionals. Pain and quality of life, for instance, emerge in some studies as more pertinent to patients, while objective measures assume greater relevance for physicians [32]. In a similar vein, the utilization of visual analogue scales could contribute to this disparity. Differences in scale presentation, anchoring, and verbal descriptors hold substantial potential to influence the resulting assessments [28, 33]. The research highlighting different aspects of disease activity for patients and physicians should be interpreted with caution, because some of the PROs capture symptoms that result from non-inflammatory elements of RA, which would not warrant treatment escalation. In other words, in some cases there may be discrepant perceptions on the part of patients or physicians about why a therapeutic target is not reached: the rheumatologist will not escalate treatment if the measured disease activity and PRO are not attributable to active inflammation, and this may contradict the patient’s expectation if they understand that all symptoms indicate disease activity and can thus be mitigated by treatment escalation. Here as always, patient education is paramount in aligning realistic expectations. Finally, it should be noted that nearly half of the rheumatologists assessed herein are concerned with insufficient financial resources to allow seeing patients frequently enough to meet T2T recommendations and with the fact that medical charts are not adapted to document PROs and measures of disease activity. With regard to the latter concern, there is evidence that the documentation of disease activity can be considerably improved by explicit attention to capturing the required information in medical records [34].

Despite the existence of barriers, Brazilian prospective studies demonstrate that the implementation of the T2T strategy for treating RA was not only feasible but also effective within the public health system. The first published cohort study in Brazil involving 241 consecutive RA patients, who were followed for 14 (±5.3) months as part of a T2T intervention, yielded promising results [35]. By the end of the follow-up period, implementation of a T2T approach led to a significantly higher proportion of patients to achieve remission according to Disease Activity Score—28 joints (DAS28; 11.6% vs. 18.6%; *p* < 0.001) and Clinical Disease Activity Index (CDAI; 8.1% vs. 13.6%; *p* < 0.001) assessments, along with low disease activity based on DAS28 (9.8% vs. 13.0%; *p* < 0.001) and CDAI (23.9% vs. 28.4%; *p* < 0.001) criteria. The incorporation of the T2T strategy into the management of RA was demonstrated to be both feasible and effective in this population, even in the context of a poor socioeconomic scenario. Furthermore, the integration of innovative therapeutic options within the framework of the T2T strategy was associated with additional improvements in disease activity and physical function, particularly for patients facing challenging disease control [35].

Importantly, the participants of the current survey reported knowledge, skill or effective action gaps related to assessing health literacy and to using tools that enlist active patient participation and patient-education resources, as well as concerns with patient adherence. On the other hand, only approximately 10% of invited rheumatologists participated in the current survey, and this is a limitation of the study. In a study conducted in Brazil, adequate health literacy was negatively associated with higher Health Assessment Questionnaire scores, whereas high activation levels were negatively associated with moderate to severe functional limitation [36]. Although concerns with patient adherence and health literacy seem to be ubiquitous in the management of patients with RA [21, 37–39], EVEREST and similar initiatives have the potential to address these and other important issues that aim at aligning healthcare providers and patients when it comes to treatment goals and understanding [38, 40]. Even though its recent use was in great part due to the coronavirus 19 pandemic, telehealth in general has great potential to enhance implementation of T2T in RA [41, 42]. For this purpose, the validation of tools for remote patient monitoring is crucial. Recently, Lineburger et al. conducted an evaluation of the cross-cultural and clinical validation of the Multidimensional Health Assessment Questionnaire/Routine Assessment of Patient Index Data 3 (MDHAQ/RAPID3) instrument in electronic format for a Brazilian population of patients with RA. The tool exhibited a 92% acceptance rate among participants. The utilization of MDHAQ/RAPID3, in conjunction with traditional clinical measures, can facilitate remote follow-up according to T2T approach, with performance comparable to the gold standard DAS28. The authors concluded that these results establish MDHAQ/RAPID3 as a viable tool for Brazilian patients with RA within the current telehealth context [43]. On the other hand, several challenges remain to effective and widespread use of telehealth in this setting, and studies such as the current one can help tailor strategies depending on local needs [41].

It should be noted that the current report presents the expert-opinion elicitation component of the first phase of EVEREST as related to Brazil. The information collected in this first phase has been used to implement the second phase, which aims to design evidence-based tools to address the identified barriers and challenges in the implementation of T2T in RA. These insights are poised to guide forthcoming stages of the EVEREST initiative both within the country and on a global scale. Such initiatives will include resources focused on three main pillars: (1) rheumatologists’ self-reflection on the use of T2T in their clinical practice; (2) shared decision-making; and (3) optimization of T2T implementation through telehealth. The third phase of EVEREST will consist of the actual use of these tools, the assessment of their contribution to T2T implementation, and their refinement according to findings.

## Electronic Supplementary Material

Below is the link to the electronic supplementary material.

Supplementary Material 1

## Data Availability

The datasets used and/or analyzed during the current study are available from the corresponding author on reasonable request.
