# Supplementary material for: Challenges in implementing treat-to-target in rheumatoid arthritis: a perspective from Brazilian rheumatologists
Source: Adv Rheumatol. 2024 Aug 26;64:63. doi: 10.1186/s42358-024-00403-w (PMC13488712; doi:10.1186/s42358-024-00403-w)
Supplement: Supplementary file 1 [file 42358_2024_403_MOESM1_ESM.docx]

Online survey

Section 1: Introduction

Obrigado pelo seu interesse! Antes de começar, leia e confirme que leu as seguintes instruções marcando a caixa à esquerda:

- Compreendo que o inquérito deverá demorar aproximadamente 5-8 minutos a concluir.
- Comprometo-me a ler atentamente cada item e a fornecer uma resposta honesta e precisa em cada um dos casos.

“Este inquérito irá demorar aproximadamente 5-8 minutos a concluir. As suas respostas irão fornecer informações para o ensino no futuro. Antes de iniciar o inquérito, certifique-se de que pode dedicar tempo suficiente para fornecer respostas sinceras e precisas. **Se necessário, pode iniciar o inquérito e completá-lo mais tarde**. Participe **apenas uma vez** no inquérito. As suas respostas devem refletir a sua atividade atual. Obrigado pela sua participação.”

Section 2: Knowledge

Question 1. **Para cada item abaixo, indique o seu nível atual de conhecimentos sobre cuidados a doentes com Artrite Reumatoide (AR)**

**Nota:** Se um item NÃO for relevante para a sua função profissional atual, assinale NÃO RELEVANTE ("NR").

1=Sem conhecimentos; 2=Conhecimentos básicos; 3=Alguns conhecimentos; 4=Bastantes conhecimentos; 5=Conhecimentos especializados; NR=Não relevante

Utilize a escala de classificação mostrada acima para classificar cada item.

**Itens de conhecimentos**

Recomendações/diretrizes internacionais para a utilização de uma estratégia "treat-to-target" (T2T) no tratamento da AR

Princípios gerais para a utilização de uma abordagem T2T em AR

Critérios onde é necessário ajustar o tratamento

Medidas compósitas para avaliar a atividade da doença

Resultados relatados pelo doente (PROs) para avaliar a atividade da doença

Como uma abordagem T2T deve ser explicada aos doentes

Princípios gerais de tomada de decisão partilhada

Métodos normalizados para avaliar os níveis de alfabetização de saúde dos doentes

Section 3: Skill

Question 2. **Para cada item abaixo, indique o seu nível atual de competências sobre cuidados a doentes com Artrite Reumatoide (AR)**

**Nota:** Se um item NÃO for relevante para a sua função profissional atual, assinale NÃO RELEVANTE ("NR").

1=Sem competências; 2=Competências básicas; 3=Algumas competências; 4=Bastantes competências; 5=Competências especializadas; NR=Não relevante

Utilize a escala de classificação mostrada acima para classificar cada item.

Itens de competências

Adaptar os princípios da T2T ao meu contexto clínico no tratamento de doentes com AR

Determinar um resultado desejado (objetivo) que é adequado para um doente específico

Realizar contagens conjuntas normalizadas

Utilizar resultados relatados pelo doente (PROs) para avaliar a atividade da doença

Ajustar o tratamento de acordo com a atividade da doença

Documentar adequadamente a atividade da doença

Prescrever medicamentos antirreumáticos modificadores da doença (DMARD)s

Prescrever e recomendar novos tratamentos aos doentes

Determinar o nível de alfabetização da saúde dos meus doentes

Section 4: Confidence

Question 3. **Para cada item abaixo, indique o seu nível atual de confiança sobre cuidados a doentes com Artrite Reumatoide (AR)**

**Nota:** Se um item NÃO for relevante para a sua função profissional atual, assinale NÃO RELEVANTE ("NR").

1=Nada confiante, 2=Algo confiante, 3=Bastante confiante, 4=Confiante, 5=Muito confiante, NR=Não relevante

Utilize a escala de classificação mostrada acima para classificar cada item.

Itens de confiança

Utilizar uma abordagem T2T para tratar doentes com artrite reumatoide

Explicar a abordagem T2T a doentes com artrite reumatoide

Explicar um ajuste de tratamento a um doente com AR gerido de acordo com a abordagem T2T

Adaptar a minha linguagem ao comunicar com doentes com AR com reduzidos níveis de alfabetização de saúde

Considerar a influência das comorbidades ao avaliar a atividade da doença

Ter os próprios objetivos dos doentes em consideração quando planeio a abordagem T2T

Section 5: Agreement (Attitudes/ Context)

Question 4. **Para cada item abaixo, indique o seu nível de concordância sobre cuidados a doentes com Artrite Reumatoide (AR)**

**Nota:** Se um item NÃO for relevante para a sua função profissional atual, assinale NÃO RELEVANTE ("NR").

0=se não tiver a certeza ou não estiver suficientemente informado para formar uma opinião ; 1=Discordo plenamente; 2=Discordo; 3=Discordo ligeiramente; 4=Concordo ligeiramente; 5=Concordo; 6=Concordo plenamente NR=Não relevante

Utilize a escala de classificação mostrada acima para classificar cada item.

Itens de concordância

No exercício da minha atividade, só utilizo medidas compósitas para avaliar a atividade da doença

A reduzida atividade da doença é uma meta de tratamento razoável, especialmente para doentes de longa duração.

As medidas de atividade da doença podem proporcionar resultados contraditórios

A perspectiva dos doentes sobre o que constitui resultados de tratamento bem sucedidos difere frequentemente das minhas próprias perspectivas

Considero um desafio monitorizar a atividade da doença

Tenho as comorbidades em consideração ao implementar a abordagem T2T

Tenho possíveis eventos adversos em consideração ao implementar a abordagem T2T

Uma estratégia T2T só é útil numa pequena parte dos meus doentes com AR

Tenho acesso a recursos e materiais informativos relevantes que posso partilhar com os meus doentes

Mantenho-me atualizado(a) relativamente a mudanças nas recomendações e diretrizes de T2T e tento adaptar a minha arividade em conformidade

Considero a tomada de decisão partilhada essencial para a abordagem T2T

No meu consultório, documento a avaliação da doença nos registos médicos ou nas fichas médicas

Colaboro e comunico com outras profissões e especialidades ao implementar a abordagem T2T em doentes

Considero um desafio identificar as preferências do doente para ajudar a determinar uma meta de tratamento adequada

Considero difícil usar a abordagem T2T em doentes em consultas de telessaúde (em modo remoto)

Confio nos relatos dos doentes comunicados pelos próprios

Contextual/ Systemic Barriers

Question 5. **Indique até que ponto os seguintes fatores são barreiras que encontra ao documentar a atividade da doença:**

**Escala de 1=Nada importante; 2=Barreira menor; 3=Barreira moderada; 4=Barreira grave; NA=Não se aplica**

A falta de medidas compósitas validadas que eu possa usar

Registos médicos ou fichas médicas não adaptados para documentar estas medidas

Avaliações contraditórias entre resultados relatados pelo doente e medidas compósitas

Demasiadas faltas do doente a consultas regulares

Mudança da atividade da doença provavelmente devido à não adesão ao tratamento por parte do doente

A minha falta de confiança nos resultados relatados pelo doente

Os processos no meu consultório/sistema de saúde não me permitem observar doentes com a frequência necessária para cumprir as recomendações T2T

Os recursos financeiros insuficientes não me permitem observar doentes com a frequência necessária para cumprir as recomendações T2T

Section 6: Behaviours

Question 6: **Classifique as diferentes considerações listadas abaixo, da mais importante para a menos importante, ao decidir ajustar o tratamento.** ( pergunta de classificação)

**Começando com 1=mais importante, classifique por ordem decrescente de importância. Selecione NA em caso de "Não Aplicável"**

| **Item** | **Classificação** |
| --- | --- |
| Resultados relatados pelo doente |  |
| Medidas compósitas |  |
| Alterações estruturais |  |
| Insuficiência funcional |  |
| Comorbidades |  |
| Preferências do doente |  |
| Nível de adesão do doente |  |
| Impacto na cobertura do plano de saúde |  |

Question 7. **Para cada item abaixo, selecione a frequência com que realiza cada uma das tarefas que se seguem nos cuidados a doentes com Artrite Reumatoide (AR)**

**Nota:** Se um item NÃO for relevante para a sua função profissional atual, assinale NÃO RELEVANTE ("NR").

1=Nunca (0%); 2=Raramente (1% -30%); 3=Ocasionalmente (30% -70%); 4=Frequentemente (70% -99%); 5=Sempre (100%) NR=Não relevante

Utilize a escala de classificação mostrada acima para classificar cada item.

Itens de comportamento

Aplico as recomendações T2T no exercício da atividade diária

Avalio e documento a medida compósita da atividade da doença

Utilizo medições quantitativas da atividade da doença

Utilizo uma calculadora de atividade da doença para calcular a pontuação da medida compósita

Utilizo aplicações/instrumentos para monitorizar a atividade da doença

Incentivo os doentes a utilizar instrumentos para monitorizar a atividade da doença

Recomendo a mudança de tratamento para doentes com atividade elevada da doença

Comunico com membros da equipa multidisciplinar para garantir a aplicação da abordagem T2T

Informo os meus doentes sobre todas as opções terapêuticas disponíveis

Compreendo as prioridades dos doentes enquanto lhes comunico as opções de mudança de tratamento

Procuro entender os motivos pelos quais os doentes recusam uma mudança de tratamento recomendada

Tenho em consideração o nível de alfabetização da saúde do doente durante a comunicação

Encaminho os doentes para materiais educacionais que os ajudarão a tomar melhores decisões

Utilizo a telessaúde para monitorizar os meus doentes

Question 8. **Com que frequência utiliza os seguintes instrumentos?**

**Nota:** Se um item NÃO for relevante para a sua função profissional atual, assinale Não relevante ("NR").

1=Nunca; 2=Raramente; 3=Ocasionalmente; 4=Frequentemente; 5=Sempre NR=Não relevante

Utilize a escala de classificação mostrada acima para classificar cada item.

a. Questionário de auto-reflexão para avaliar o exercício da minha atividade clínica

b. Lista de verificação de considerações de tratamento para apoiar a minha escolha de tratamento

c. Lista de verificação de comorbidades para considerar possíveis comorbidades da AR

d. Cenários para me ajudar a melhorar a minha tomada de decisão crítica

e. Discussões da equipa multidisciplinar do caso para melhorar a colaboração com outras especialidades

f. Lista de verificação de intervenções não farmacológicas que podem ser adaptadas às necessidades individuais do doente

g. Instrumentos de tomada de decisão partilhadas

h. Recursos educacionais para o doente

Question 9. (If Q9, a to f = 1=Never; 2=Rarely; )**Por que não utiliza este instrumento? (selecione todas as opções aplicáveis)**

Na medida dos meus conhecimentos, não existe qualquer instrumento

Demorado

Não preciso dos instrumentos

Não cumpre o seu objetivo

Não é adequado aos meus doentes

É de difícil utilização

Falta de clareza

Demasiado simplificado

Não está disponível no meu idioma local

Dispendioso

Não se adapta ao meu contexto cultural
